# High-Incidence of Human Adenoviral Co-Infections in Taiwan

**DOI:** 10.1371/journal.pone.0075208

**Published:** 2013-09-20

**Authors:** Shan-Li Wang, Chia-Yu Chi, Pin-Hwa Kuo, Huey-Pin Tsai, Shih-Min Wang, Ching-Chuan Liu, Ih-Jen Su, Jen-Ren Wang

**Affiliations:** 1 National Institute of Infectious Diseases and Vaccinology, National Health Research Institutes, Tainan, Taiwan; 2 Department of Pathology, National Cheng Kung University Hospital, Tainan, Taiwan; 3 Department of Emergency Medicine, National Cheng Kung University Hospital, Tainan, Taiwan; 4 Department of Medical Laboratory Science and Biotechnology, College of Medicine, National Cheng Kung University, Tainan, Taiwan; 5 Center of Infectious Disease and Signaling Research, National Cheng Kung University, Tainan, Taiwan; 6 Department of Pediatrics, College of Medicine, National Cheng Kung University, Tainan, Taiwan; 7 Department of Emergency Medicine, College of Medicine, National Cheng Kung University, Tainan, Taiwan; University of Rochester Medical Center, United States of America

## Abstract

**Background:**

Respiratory infections caused by adenovirus (HAdV) are common year round. Recently, a significant increase of adenoviral infections was observed in Taiwan.

**Objective:**

To understand the prevalence and molecular epidemiology of respiratory adenovirus circulating in Taiwan for the past decade.

**Study Design:**

One hundred and twenty-six human adenoviruses, isolated between 2002 to 2011, were characterized via DNA sequencing of the hexon and fiber genes. The nucleotide sequences were then compared by phylogenetic analysis.

**Results:**

HAdV-B3 accounted for 64.3% (81/126) and peaked almost every year, whereas the sequences of hexon and fiber genes of HAdV-B3 were highly conserved in different years. A high incidence of co-infection of adenoviruses was observed (19.0%, 24/126); HAdV-B3 co-infected with HAdV-C2 was the most common combination (58.3%, 14/24). An additional interesting finding of repeated infection was noted in 10 children, all of whom showed first infection with adenovirus species HAdV-C, followed by species HAdV-B or HAdV-E.

**Conclusions:**

HAdV-B3 was the predominant type of respiratory adenovirus circulating in Taiwan over the past ten years. This merits further attention for vaccine development. Furthermore, the observed high-incidence of adenoviral co-infections along with repeated infections found in our study provides important epidemiological insights into adenovirus infections.

## Introduction

Adenoviruses exhibit various clinical presentations: respiratory illness, gastroenteritis, ocular or urinary tract infection [1-6]. Respiratory adenoviruses usually cause mild upper but can also cause severe lower respiratory tract infection [7,8]. Our prior report showed adenovirus accounting for about 4.0% of all respiratory infections in Taiwan during 1997-1999 and was the most common agent associated with tonsillitis and pharyngitis [9].

Adenoviruses are non-enveloped DNA viruses and are divided into seven species (A to G) with more than 57 genotypes identified [10]. In general, respiratory disease-associated adenoviruses include species HAdV-B (HAdV-B3, HAdV-B7, HAdV-B11 and HAdV-B14), HAdV-C (HAdV-C1, HAdV-C2 and HAdV-C5) and HAdV-E (HAdV-E4) [11-14]. Molecular epidemiology of respiratory adenoviruses, assessed by DNA sequencing of the hexon and/or fiber genes, has been reported in several countries. In the USA (2007-2008), Germany (2001-2007), Palestine (2005-2010) and China (2006-2008), HAdV-B3 was the most predominant type, followed by HAdV-B7 or HAdV-C2 [15-18]. In contrast, Israel (2006-2008) and Malaysia (1999-2005) had HAdV-C1 and HAdV-C2 as predominant types, respectively [19,20]. By using genomic-RFLP or PCR-RFLP analysis, previous reports have shown that HAdV-E4 and HAdV-B3 were the major types of respiratory adenovirus circulating in southern Taiwan in 2001 and 2002 respectively [21]. HAdV-B3 was the major type circulating in northern Taiwan during 2004 and 2005 [22].

Most recently, a significant increase of adenovirus prevalence was found in Taiwan in 2011, possibly due to different emerging adenoviruses. To better understand the molecular epidemiology and evolution of adenoviruses circulating in Taiwan, we investigated the genotypes of respiratory adenoviruses circulating in Taiwan over a full decade (2002 to 2011) by DNA sequencing of hexon and fiber genes. The prevalent types of adenovirus in Taiwan in the past decade were identified. Interestingly, a high-incidence of adenoviral co-infections as well as adenoviral repeated infections was revealed in this study.

## Materials and Methods

### Ethics Statement

This study was conducted in Taiwan only, and Institutional Review Board (IRB) approval was obtained from National Cheng Kung University Hospital (No. A-BR-101-020). This was a retrospective study without intervention or obtaining extra clinical specimens. Human specimens were not directly used in this research and informed consent was waived. The waiving of informed consent was also approved by the Institutional Review Board of National Cheng Kung University Hospital.

### Viral culture

Viral cultures of laboratory-confirmed adenovirus infection were processed as described previously [9]. This study used adenovirus isolated from either nasopharyngeal aspirate or throat swabs; and stored at -80°C at the Virology Laboratory of National Cheng Kung University Hospital before use. The study population was children from either hospitalized patients or outpatients of Cheng Kung University Hospital. Viruses used in this study were sub-cultured in A549 cells with Dulbecco’s modified eagle medium (DMEM) supplemented with 10% fetal bovine serum (FBS), 100 U/ml penicillin and 100 μg/ml streptomycin. A549 cells in 15-ml culture tubes were infected with viral suspension, and then incubated at 35°C with 5% CO_2_. When 85% cytopathic effect was observed, cells were harvested for DNA extraction.

### Viral genome extraction and genotyping

Viral DNA was extracted by the phenol/chloroform method according to a modified procedure from a previous report [23]. Volumes of reagents used in the procedure were reduced, and DNA pellets were finally resuspended in 50 μl of ddH_2_O.

To type the adenovirus, the Loop 1 region of the hexon gene was amplified with generic primer pair HXL1F (5′-CGTGTGCAGTTYGCCCG) and HXL1R (5′-ACAGCCTGATTCCACAT). The Loop 2 region of the hexon gene was amplified with generic primer pair BL (5′-TTGACTTGCAGGACAGAAA) and BR (5′-CTTGTATGTGGAAAGGCAC) (Table S1) [24]. To identify the hexon gene in co-infected isolates, type-specific primer pairs were designed. For the Loop 2 region, primers AdC2F and HXL2R were used for HAdV-C2; AdC5F and HXL2R for HAdV-C5; CDL and HXL2R-2 for HAdV-C6. In addition, AdBL1F and BR were used to amplify the region containing both Loop 1 and 2 for HAdV-B3. To amplify the fiber gene, primers FiBL and FiBR were used for HAdV-B3, HAdV-E4, HAdV-B7 and HAdV-B11; FiCL-2 and FiCR-2 for HAdV-C1; FiCL and FiCR for HAdV-C2; FiCL-2 and FiCR-3 for HAdV-C5. PCR mixtures consisted of 1 U of DNA polymerase (KOD plus polymerase, Toyobo), 1 mM MgSO_4_, 0.2 mM dNTP, 300 pM of each primer and 1 to 2 μl of template from the original purified DNA solution in a 50-μl reaction volume. PCR cycling consisted of initial denaturation at 95°C for 5 minutes, followed by 40 cycles of 94°C for 20 seconds, 54°C or 56°C for 20 seconds, depending on primers used, and 72°C for 40 or 80 seconds, depending on the length of PCR products. DNA sequencing of PCR products was carried out with the primers used in PCR by Sanger’s method.

### Phylogenetic analysis

Phylogenetic trees were constructed by the neighbor-joining method based on PAUP software [25]. About 1500 base pairs of hexon gene (nt 139-1612 based on HAdV-B3) and 700 base pairs of fiber gene (nt 224-888 based on HAdV-B3) were analyzed. Sequences of reference strains were obtained from GenBank (for hexon gene, accession number for HAdV-C1 (Adenoid 71): AB330082; HAdV-C2 (Adenoid 6): AB330083; HAdV-B3 (837-05-TW): EF494650; HAdV-E4 (NHRC 3): AY599837; HAdV-C5 (Adenoid 75): AB330086; HAdV-C5 (RKI-4592/07): EU867493; HAdV-C6 (Tonsil 99): HQ413315; HAdV-B7 (1106/SJZ/CHN/2001): JQ360622; HAdV-B11 (Taiwan/760/2002): FJ841905; HAdV-B16 (ch. 79): AY601636). Sequences identified were submitted under GenBank accession number KC570845 to KC570909. Nucleotide comparisons of hexon and fiber genes were calculated using the DNA distance matrix program in BioEdit.

## Results

### DNA sequencing of hexon and fiber genes of respiratory adenovirus circulating between 2002 and 2011

**Figure 1:**
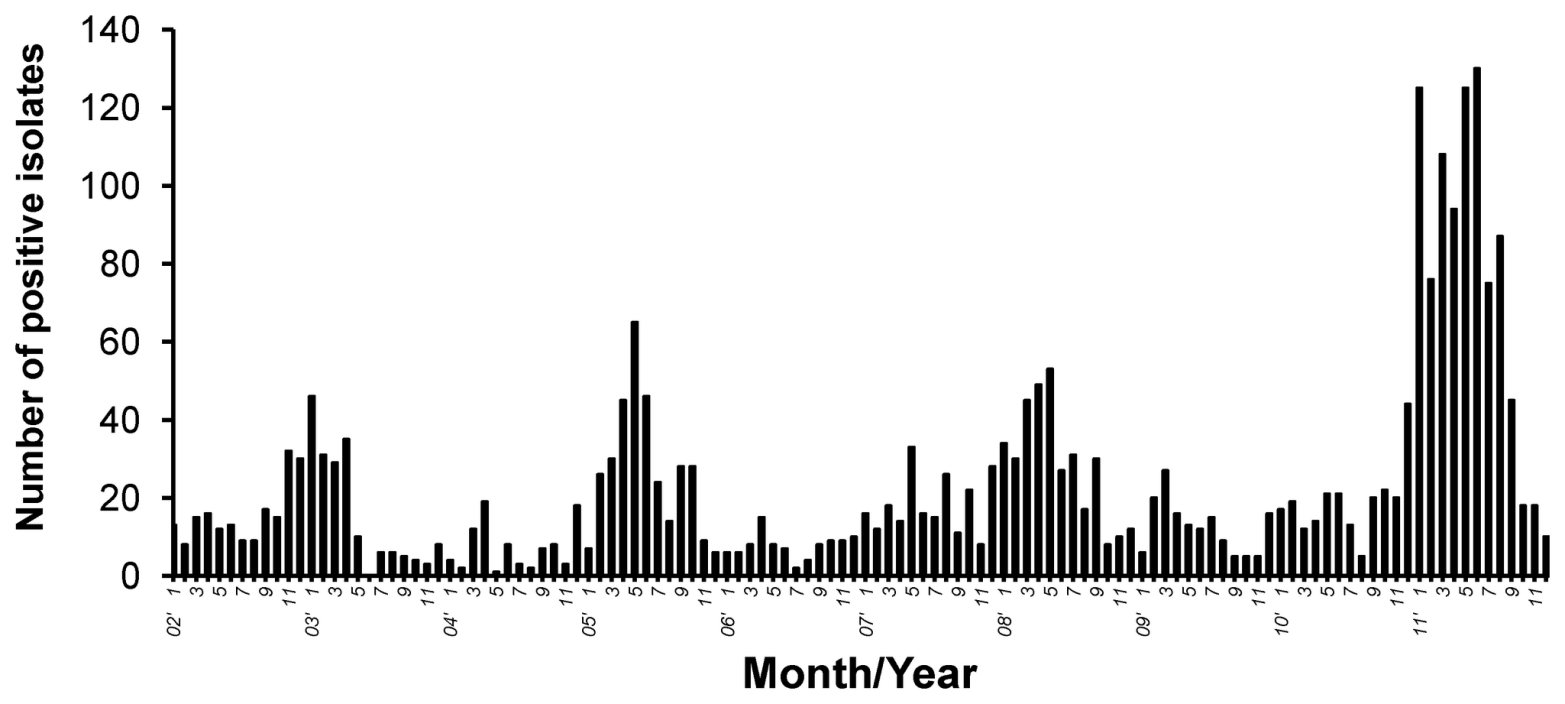
Numbers of respiratory adenoviral isolates collected at National Cheng Kung University Hospital from 2002 to 2011.

**Figure 2:**
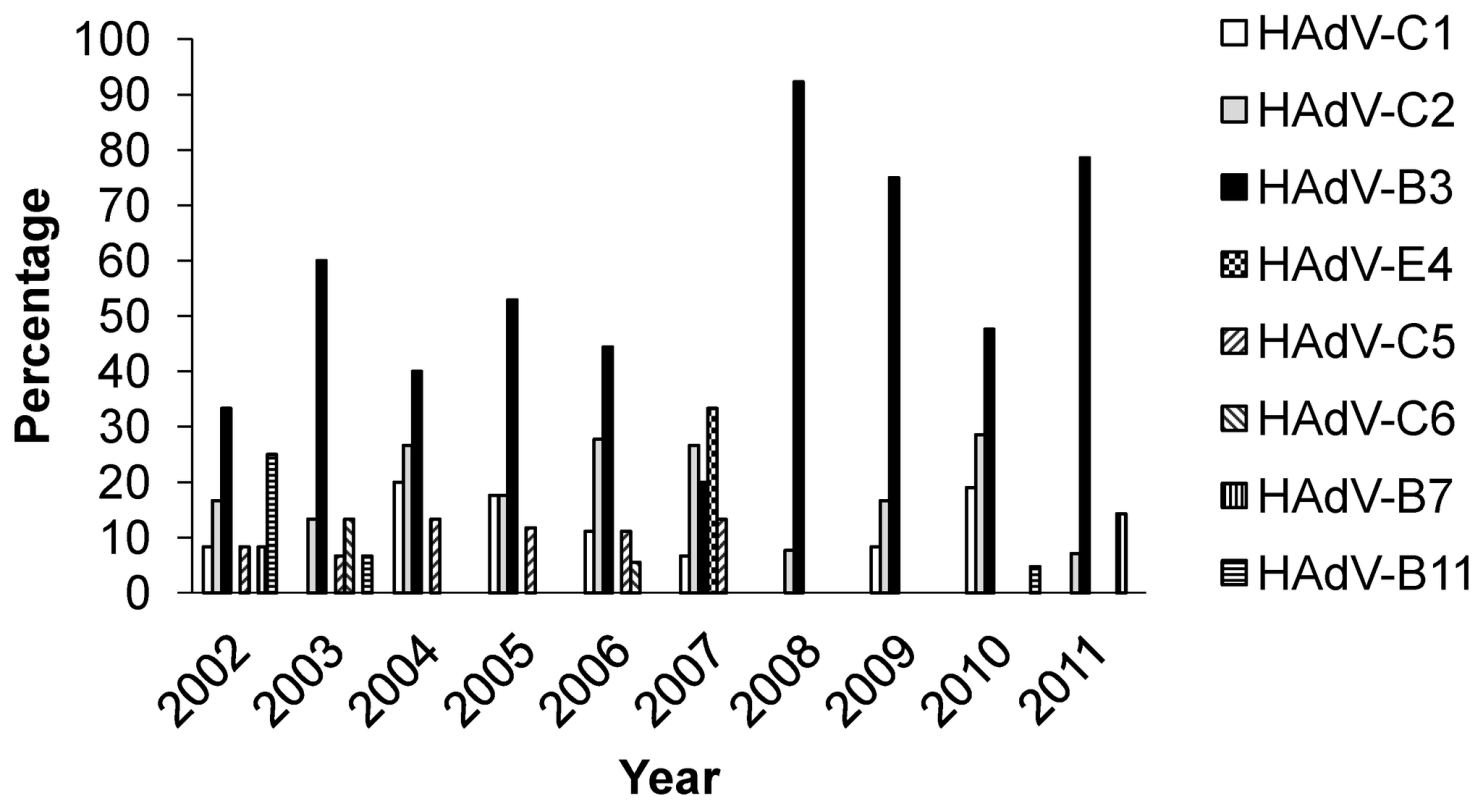
Proportional distribution of different adenoviral types from 2002 to 2011. Total amount of HAdV-C1, HAdV-C2, HAdV-B3, HAdV-E4, HAdV-C5, HAdV-C6, HAdV-B7 and HAdV-B11 strains is 15, 30, 81, 5, 10, 3, 3 and 5, respectively.

To analyze the molecular epidemiology of respiratory adenoviruses circulating in Taiwan, adenovirus-positive isolates collected at National Cheng Kung University Hospital (NCKUH) during 2002-2011 were examined. There were 2,001 adenovirus-positive isolates associated with respiratory diseases (Figure 1). One to two isolates per month over a decade were randomly selected for our investigation. A total of 106 adenovirus isolates were successfully subcultured. In addition, 20 isolates from repeated infections were also examined. The repeated infections were identified from our adenovirus database by computerized system according to patient identification number. In total, there were 126 isolates from 52 females and 74 males. Median age of selected patients was 3.9 years (range: 8 months to 9.3 years). Predominant clinical features of selected patients included tonsillitis, pharyngitis and bronchopneumonia (34.6, 19.3 and 16.1%, respectively). Viral genomes of 126 isolates were extracted, partial hexon gene sequences amplified by PCR and then sequenced. Primer pairs targeting Loop 1 (nucleotides 82-1151 based on HAdV-B3) or Loop 2 (nucleotides 1055-1660 based on HAdV-B3) regions of hexon gene were used and fiber gene partial sequence assessed. Specific primer pairs corresponding to fiber gene types were designed for use in PCR and sequencing. A total of 152 strains belonging to eight types were identified from 126 isolates, including HAdV-B3, HAdV-B7, HAdV-B11, HAdV-C1, HAdV-C2, HAdV-C5, HAdV-C6 and HAdV-E4. HAdV-B3 (64.3%, 81/126) was predominant, followed by HAdV-C2 (23.8%, 30/126). Yearly distribution of types showed adenovirus HAdV-B3 continually appearing from 2002 to 2011, peaking in every year except 2007 (Figure 2). HAdV-C2 also continually appeared with less activity than HAdV-B3 from 2002 to 2011; HAdV-E4 only appeared and peaked in 2007.

### Multiple infections of adenovirus

**Figure 3:**
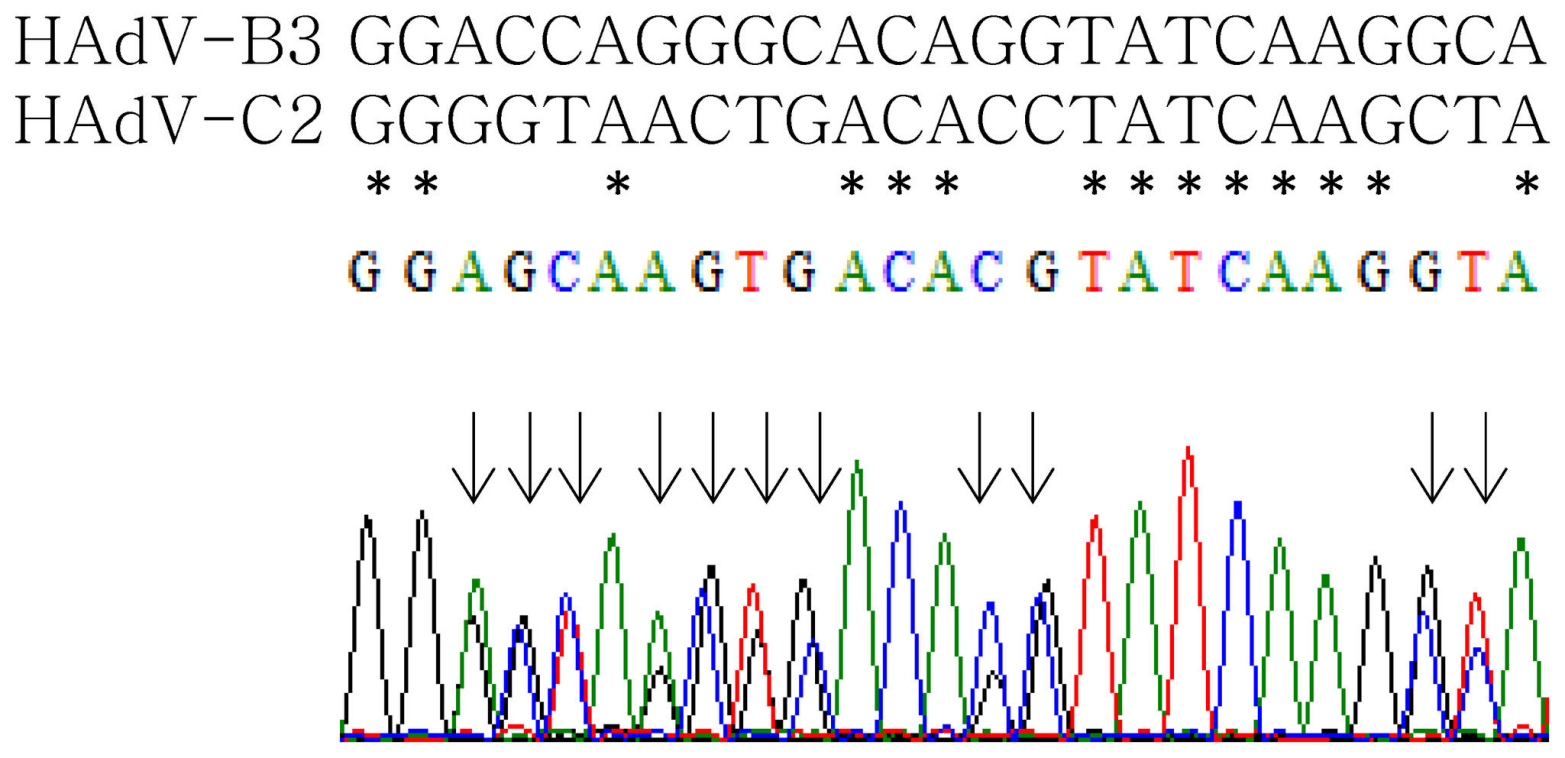
Multiple templates revealed after nucleotide sequencing of the hexon Loop 2 region. Partial result of the chromatogram of N8630/TW/09 is shown, sequencing carried out with the primer BL used in the PCR. Upper panel shows nucleotide sequences of HAdV-B3 (1240-1264) and HAdV-C2 (1297-1321) corresponding to the region, with consensus nucleotides marked. The phenomenon of multiple templates was also observed when nucleotide sequencing was carried out with primer BR (data not shown).

Sequencing results showed superimposed peaks in the chromatograms in 26 isolates using the generic primers BL and BR for the hexon gene (Figure 3). Multiple templates indicated a mix of multiple adenoviral strains in one specimen. To confirm this probability, PCR products amplified by BL and BR for hexon Loop 2 region in two co-infected isolates were cloned and sequenced; distinct hexon genes of different types were verified (data not shown). The other co-infected isolates were confirmed by direct evidence of sequencing results of the hexon and/or fiber genes using type-specific primers developed in this study for different hexon (Loop 1 or 2) or fiber regions (Table S1). Co-infections were confirmed when two types of hexon genes and/or fiber genes from the same region were identified by sequencing (Table 1). In all, 24 samples (19.0%, 24/126) with more than one adenoviral strain in each were identified: 14 samples (58.3%) containing HAdV-C2 and HAdV-B3; 5 (20.8%) containing HAdV-C1 and HAdV-B3; 2 (8.3%) containing HAdV-B3 and HAdV-C5; 1 each (4.1%) containing HAdV-B3 and HAdV-C6; HAdV-C2 and HAdV-B11; and HAdV-C2, HAdV-B3 and HAdV-B11. Interestingly, all co-infected strains involved different species of adenovirus, including species HAdV-B and HAdV-C.

**Table 1:** The co-infection isolates found in this study ^a^.

Isolate	Hexon	Fiber	Isolate	Hexon	Fiber
N625/TW/02	**HAdV-C2**, HAdV-B3	HAdV-C2	N4138/TW/05	**HAdV-C1**, HAdV-B3	HAdV-C1, HAdV-B3
N916/TW/02	**HAdV-C1**	HAdV-C1, HAdV-B3	N586/TW/06	**HAdV-C2**	HAdV-C2, HAdV-B3
N1283/TW/02	**HAdV-C2**, HAdV-B3	HAdV-C2, HAdV-B3	N920/TW/06	HAdV-B3, **HAdV-C5**	HAdV-B3, HAdV-C5
N78/TW/03	**HAdV-C2**, **HAdV-B3**	HAdV-C2, HAdV-B3	N1932/TW/06	**HAdV-C2**	HAdV-C2, HAdV-B3
N1184/TW/03	**HAdV-C2**, HAdV-B11	HAdV-C2, HAdV-B11	N68/TW/07	**HAdV-C2**	HAdV-C2, HAdV-B3
N1616/TW/03	HAdV-B3, **HAdV-C5**	HAdV-B3, HAdV-C5	N6125/TW/09	**HAdV-C2**, **HAdV-B3**	HAdV-C2, HAdV-B3
N3269/TW/03	**HAdV-B3**, **HAdV-C6**	HAdV-B3	N8630/TW/09	**HAdV-C2**, **HAdV-B3**	HAdV-C2, HAdV-B3
N969/TW/04	**HAdV-C1**, HAdV-B3	HAdV-C1, HAdV-B3	N83/TW/10	**HAdV-C2**, **HAdV-B3**, HAdV-B11	HAdV-C2, HAdV-B3
N3006/TW/04	**HAdV-C1**	HAdV-C1, HAdV-B3	N1210/TW/10	**HAdV-C2**, HAdV-B3	HAdV-C2, HAdV-B3
N961/TW/05	**HAdV-C2**	HAdV-C2, HAdV-B3	N3974/TW/10	**HAdV-C2**	HAdV-C2, HAdV-B3
N3573/TW/05	**HAdV-C2**, HAdV-B3	HAdV-C2, HAdV-B3	N8157/TW/10	**HAdV-C1**	HAdV-C1, HAdV-B3
N3773/TW/05	**HAdV-C2**, HAdV-B3	HAdV-C2, HAdV-B3	N15257/TW/10	**HAdV-C2**	HAdV-C2, HAdV-B3

a For hexon gene, types in **boldface** indicate that both Loop 1 and 2 were identified, and types in regular expression indicate only Loop 2 identified.

### Repeated infections of adenoviruses

From our database, we noted that there were some children with repeated adenoviral infections from 2002 to 2011. Repeated infections may be caused by a variant strain of adenovirus. Therefore, these twenty isolates from three females and seven males were examined for genotypes (Table 2). Intervals between infections in each patient ranged from three months to five years. Intriguingly, all these children were first infected with species HAdV-C adenoviruses (HAdV-C1, HAdV-C2 or HAdV-C5), followed by species HAdV-B (HAdV-B3) or species HAdV-E (HAdV-E4). This is the first report tracking repeated adenoviral infections over a decade.

**Table 2:** Repeated infections of adenovirus found in this study.

Birthday	Sex	Interval of two infections (year)	Isolate	Collected day	Type
2002 Jul 6	M	2.6	N1334/TW/05	2005 May 3	HAdV-C5
			N508/TW/08	2008 Jan 31	HAdV-B3
2002 Sep 17	M	2.0	N2020/TW/05	2005 Jun16	HAdV-C1
			N2808/TW/07	2007 Jul 2	HAdV-E4
2002 Sep 17	F	1.1	N1232/TW/06	2006 Arp 24	HAdV-C2
			N2827/TW/07	2007 Jul 3	HAdV-E4
2003 Oct 10	F	1.1	N1069/TW/07	2007 Mar 1	HAdV-C2
			N2167/TW/08	2008 May 5	HAdV-B3
2003 Dec 6	M	1.5	N/585/TW/07	2007 Jan 23	HAdV-C5
			N4884/TW/08	2008 Sep 4	HAdV-B3
2004 Dec 21	M	0.2	N2895/TW/11	2011 Feb 22	HAdV-C2
			N6829/TW/11	2011 May23	HAdV-B3
2005 Sep 18	F	5.0	N1897/TW/06	2006 Jun 12	HAdV-C5
			N9451/TW/11	2011 Jul 28	HAdV-B3
2006 Sep 30	M	2.1	N3920/TW/08	2008 Jul 14	HAdV-C2
			N13744/TW/10	2010 Sep 27	HAdV-B3
2007 Jun 5	M	1.0	N3174/TW/10	2010 Mar 18	HAdV-C1
			N4746/TW/11	2011 Mar 29	HAdV-B3
2007 Aug 7	M	0.4	N87/TW/10	2010 Jan 4	HAdV-C2
			N6429/TW/10	2010 Jun 10	HAdV-B3

### Phylogenetic analysis of the hexon and fiber genes

**Figure 4:**
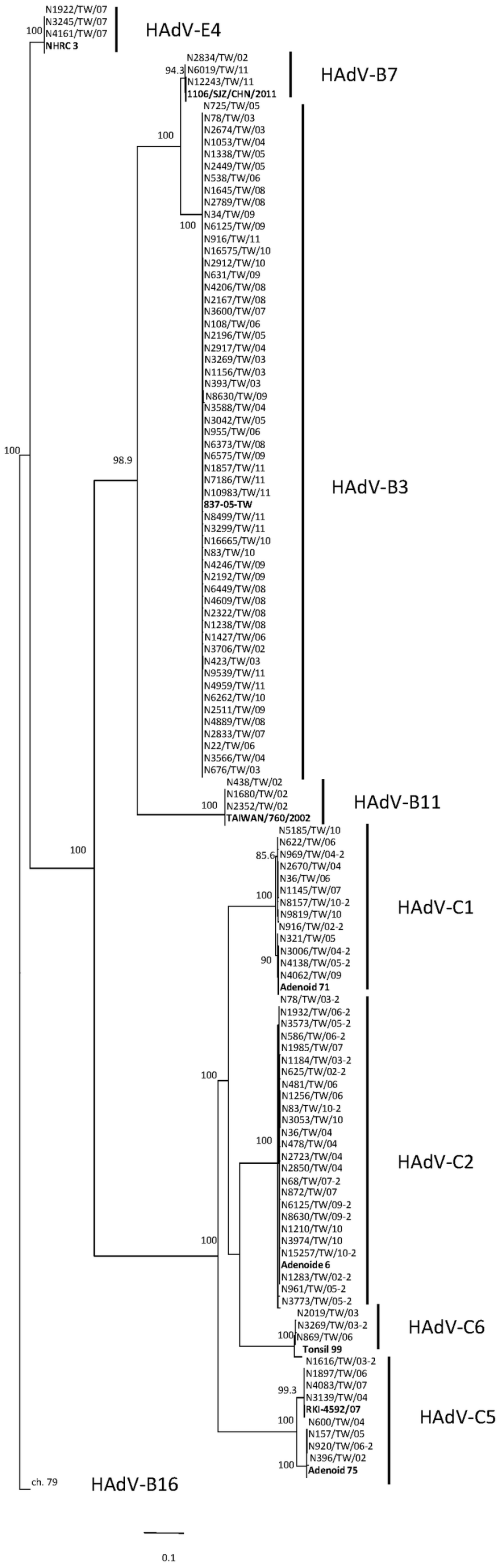
Analysis of partial hexon nucleotide sequences (nucleotides 139-1612/HAdV-B3). Phylogenetic tree with 1000 bootstrap replicates was constructed from selected sequences of adenoviruses isolated during 2002-2011, reference strains selected from GenBank. Bootstrap values greater than 80 were considered as significant and indicated in the figure. Strains of species HAdV-B from co-infected isolates are indicated by original isolate name; strains of species HAdV-C from co-infected isolates are indicated by isolate name -2 (e.g., N78/TW/03 is HAdV-B3, N78/TW/03-2 is HAdV-C2). Strains of repeated infections and strains in which only Loop 2 sequence has been identified were not shown in this phylogenetic tree.

Phylogenetic analysis of hexon and fiber genes identified 152 strains clustering with reference strains from the GenBank (Figure 4 and Figure S1). Strains of most genotypes from diverse years were grouped into only one cluster in both hexon and fiber genes, indicating sequences conserved throughout. Interestingly, only HAdV-C5 strains revealed two phylogenetic clusters with 4.7% difference in the hexon region (Table S2); by contrast, their fiber gene regions were grouped in one cluster. Differences in the fiber gene of HAdV-C1 strains of 2.2% and 6.3% were also found in nucleotide and amino acid sequences, respectively. Overall, the results indicate higher nucleotide variability in species HAdV-C adenoviruses than in species HAdV-B and HAdV-E.

## Discussion

This molecular epidemiology study of adenovirus in Taiwan found a high incidence of adenoviral co-infection along with repeated infections. Adenoviral co-infections found in the present study were based on DNA sequencing of hexon and fiber genes, thus providing a high level of confidence in the validity of our data. Furthermore, unique infection patterns involving different species and type order were revealed in repeated adenoviral infections. Taken together, our investigation affords interesting insights into the respiratory adenoviral epidemiology.

Multiple-infection (co-infection) of adenoviruses was detected in 19.0% of clinical isolates analyzed. Adenoviral co-infection has also been reported in other countries, with a range of 1.5-19.0% [14,26-30]. Variance in co-infection rate may arise from different communities where samples were taken or methodologies used. Primers used in PCR may also play a critical role in detecting co-infection. In this study, the phenomenon of multiple templates was detected in 26 isolates with the generic primer pair of hexon Loop 2, but only in two isolates with the generic primer pair of hexon Loop 1 (data not shown). This indicates that multiple primer sets may be required to detect co-infection.

This study discovered adenoviral co-infection via multiple PCR-sequencing templates, which were amplified using generic primers of the hexon Loop 2 region. Distinct hexon genes in co-infected isolates were then verified with type-specific primers and followed by nucleotide sequencing. In other studies, most adenoviral co-infections were determined with type-specific primers without DNA sequencing [14,26,27,29-31]. For detecting co-infection, the application of generic primers is more convenient than that of type-specific primers in a community with diverse adenovirus populations; nucleotide sequencing must be determined subsequently. Genotypes in co-infected isolates still must be identified with type-specific primers, since multiple templates may appear in nucleotide sequencing. Furthermore, direct sequencing of the same genomic region should be the required criterion for the determination of co-infection since recombinants between different adenoviruses may exist. In addition, our evidence for co-infections may give rise to potential recombinants which can lead to more potent HAdVs [32].

Adenoviral co-infection has never been reported in previous epidemiology studies in Taiwan, which used genomic-RFLP or PCR-RFLP for adenovirus typing [21,22]. Our study revealed only dominant type (species HAdV-C) in the genomic-RFLP pattern (data not shown). Bias in titer in co-infected isolates was also found by plaque purification in another study [31], which suggests that PCR-sequencing for genotyping may be an advantageous strategy for detecting co-infection events. Most common adenoviral co-infections revealed in this study was HAdV-B3 co-infected with HAdV-C2, likely because HAdV-B3 and HAdV-C2 are predominant types in Taiwan for the period examined. Most common co-infections of adenoviruses in other nations also correlated with the predominant types in their community [14,26,30,31], suggesting adenovirus co-infection is not type-dependent. Nevertheless, we noted that strains in co-infected isolates always came from different species (HAdV-B and HAdV-C), with the exception of one co-infected case with three strains. These findings suggest a requirement for different species involved in co-infection and warrant further investigation.

Notably, repeated infections of adenovirus from 10 children were found and analyzed; all 10 were first infected with species HAdV-C, followed by species HAdV-B or HAdV-E. This suggests that primary infection with species HAdV-C may be able to protect against subsequent HAdV-C infections, but not against subsequent infection by species HAdV-B or HAdV-E. This may also explain why the proportion of species HAdV-C adenovirus is lower than species HAdV-B in many countries [15-18]. Adenoviral infection of vaccinated persons has been reported in American military recruits [31]. Infections in vaccinated persons were usually caused by a variant of the vaccine-targeted strain HAdV-E4 or HAdV-B7, indicating that adaptive immunity induced by species HAdV-B or HAdV-E is insufficient to safeguard against infection by variant strains of species HAdV-B or HAdV-E. Repeated detection of adenovirus in upper respiratory infections has also been reported by Kula et al [33]. Three types of repeated infections were identified including 1) adenovirus of same strain, 2) adenovirus of same type but variant strains, 3) adenovirus of different types which were infected with species HAdV-B or HAdV-E (HAdV-B3 or HAdV-E4) and followed by species HAdV-C (HAdV-C1) of adenovirus in an 8-month or 24-month old child, respectively. The difference between this study and our results may be due to the younger age of patients and shorter interval of repeated infections in the study by Kula et al [33]. Taken together, these results suggest that further serologic study is required to understand the phenomenon of repeated infections of adenovirus.

This study showed HAdV-B3 as the most common respiratory adenovirus strain circulating in Taiwan during 2002-2011, except in 2007. It was likewise the most common in America, Germany, Palestine and China during 1999-2010 [15-18]. This indicates that the most prevalent type of respiratory adenovirus in Taiwan is similar to that in many other countries. Sequences of hexon and fiber genes of HAdV-B3 were highly conserved between outbreaks, suggesting HAdV-B3 outbreaks may not be caused by changes in antigenicity. Conservation of the hexon gene evident in this study is consistent with a previous report that the hexon gene was conserved in most types circulating in Japan from 1988 to 2007 [34]. This study found two phylogenic clusters of HAdV-C5 in the hexon gene. Variation within HAdV-C5 was also discovered in Germany and Japan [15,34]. It is not clear whether different HAdV-C5 clusters are prevalent in different years, since only 10 isolates of HAdV-B5 were identified here.

In summary, this 10-year surveillance study showed that HAdV-B3 accounted for 64.3% of respiratory adenoviral infections, with strong nucleotide sequence conservation of the hexon and fiber genes of HAdV-B3. The results suggest development of HAdV-B3 vaccine is warranted. In addition, a high incidence of adenoviral co-infection was revealed. Most co-infections were HAdV-B3 co-infected with HAdV-C2. Finally, variant antigenic specificity among types of adenovirus was observed in children with repeated infections.

## Supporting Information

Figure S1 **Analysis of nucleotide sequences of a portion of the fiber gene (nucleotides 224-888/HAdV-B3).** Phylogenetic tree with 1000 bootstrap replicates was constructed from selected sequences of adenoviruses isolated during 2002-2011. Reference strains were selected from the GenBank, bootstrap values greater than 80 considered as significant and indicated in the figure (Accession number for HAdV-C1: AF534906, KNIH 00/2: AY224420, Guangzhou01: DQ099432.4, NHRC 3: AY599837, HAdV-C5: AY339865, 0901HZ/ShX/CHN/2009: JF800905, Taiwan/760/2002: FJ841913). Strains of species HAdV-B from co-infected isolates are indicated by original isolate name; strains of species HAdV-C from co-infected isolates are indicated by isolate name-2 (e.g., N78/TW/03 is HAdV-B3, N78/TW/03-2 is HAdV-C2). Strains of repeated infections were not shown in this phylogenetic tree.
(TIFF)

Table S1 **Primers used in this study^a^.** ^a^ Primer BL, BR, CDL, FiBL and FiBR were designed by Madich et al. (24). ^b^ Y refers to nucleobase cytosine or thymine. ^c^ R refers to nucleobase adenine or guanine.
(DOCX)

Table S2 **Pairwise comparison of nucleotide and amino acid sequences^a^.** ^a^ About 1,500 nucleotides of hexon and 940 nucleotides of fiber genes from different types were analyzed. ^b^ NA, not available.
(DOCX)
