# Supplementary figures and images for: High-Incidence of Human Adenoviral Co-Infections in Taiwan
Source: PLoS One. 2013 Sep 20;8(9):e75208. doi: 10.1371/journal.pone.0075208 (PMC3779158; doi:10.1371/journal.pone.0075208)

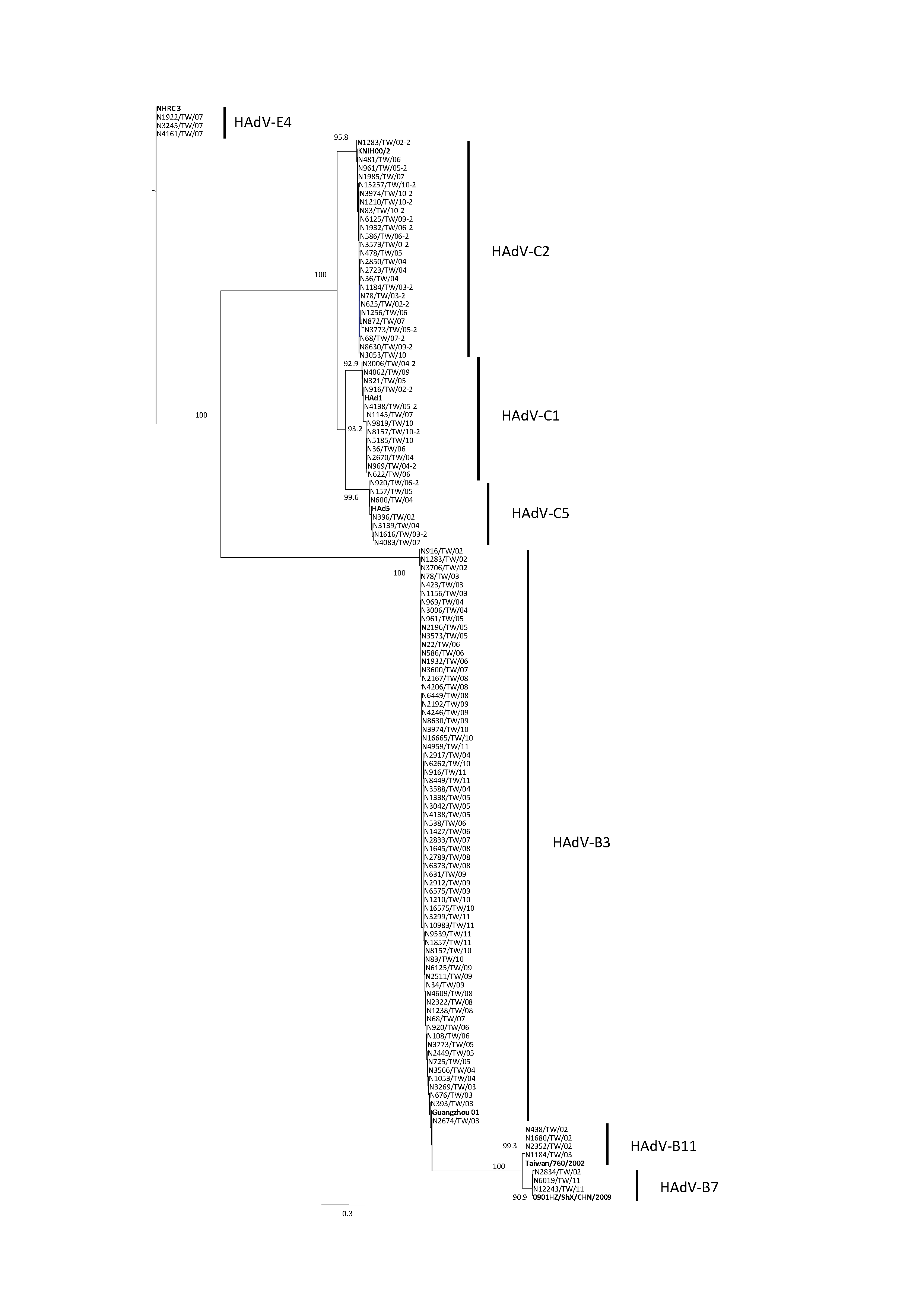

Supplement: Figure S1 — Analysis of nucleotide sequences of a portion of the fiber gene (nucleotides 224-888/HAdV-B3). Phylogenetic tree with 1000 bootstrap replicates was constructed from selected sequences of adenoviruses isolated during 2002-2011. Reference strains were selected from the GenBank, bootstrap values greater than 80 considered as significant and indicated in the figure (Accession number for HAdV-C1: AF534906, KNIH 00/2: AY224420, Guangzhou01: DQ099432.4, NHRC 3: AY599837, HAdV-C5: AY339865, 0901HZ/ShX/CHN/2009: JF800905, Taiwan/760/2002: FJ841913). Strains of species HAdV-B from co-infected isolates are indicated by original isolate name; strains of species HAdV-C from co-infected isolates are indicated by isolate name-2 (e.g., N78/TW/03 is HAdV-B3, N78/TW/03-2 is HAdV-C2). Strains of repeated infections were not shown in this phylogenetic tree. (TIFF) [file pone.0075208.s001.tiff]
