# Supplementary material for: High-Incidence of Human Adenoviral Co-Infections in Taiwan
Source: PLoS One. 2013 Sep 20;8(9):e75208. doi: 10.1371/journal.pone.0075208 (PMC3779158; doi:10.1371/journal.pone.0075208)
Supplement: Table S1 — Primers used in this studya. a Primer BL, BR, CDL, FiBL and FiBR were designed by Madich et al. (24). b Y refers to nucleobase cytosine or thymine. c R refers to nucleobase adenine or guanine. (DOCX) [file pone.0075208.s002.docx]

Table S1. Primers used in this study ^a^.

| Primer | Sequence | Region | Target type(s) | Positions/ type for positions |
| --- | --- | --- | --- | --- |
| BL | TTGACTTGCAGGACAGAAA | Hexon Loop 2 | Common | 1055-1073/HAdV-B3 |
| BR | CTTGTATGTGGAAAGGCAC | Hexon Loop 2 | Common | 1660-1642/HAdV-B3 |
| HXL1F | CGTGTGCAGTTYGCCCG^b^ | Hexon Loop 1 | Common | 82-98/HAdV-B3 |
| HXL1R | ACAGCCTGATTCCACAT | Hexon Loop 1 | Common | 1151-1135/HAdV-B3 |
| AdC2F | GGGGTGCCTCTTCCAAAG | Hexon Loop 2 | HAdV-C2 | 779-816/HAdV-C2 |
| HXL2R | CTGAAGTTCCACTCRTA^c^ | Hexon Loop 2 | HAdV-C2, HAdV-C5 | 1802-1789/HAdV-C2 |
| AdC5F | CAACCATTAAGGAAGGTA | Hexon Loop 2 | HAdV-C5 | 911-928/HAdV-C5 |
| CDL | GTTGACTTGCAAGACAGAAA | Hexon Loop 2 | HAdV-C6 | 1099-1118/HAdV-C6 |
| HXL2R-2 | CTGAAGTTGCATTCATAT | Hexon Loop 2 | HAdV-C6 | 1790-1773/HAdV-C6 |
| AdBL1F | CAGTATGGGGAACAAATTTAGA | Hexon Loop 1 | HAdV-B3 | 117-138/HAdV-B3 |
| FiBL | TACCCCTATGAAGATGAAAGCA | Fiber | HAdV-B3, HAdV-E4, HAdV-B7, HAdV-B11 | 43-63/HAdV-B3 |
| FiBR | GGAGGCAAAATAACTACTCG | Fiber | HAdV-B3, HAdV-E4, HAdV-B7, HAdV-B11 | 1028-1009/HAdV-B3 |
| FiCL | GACCGTCTGAAGACACCTTCA | Fiber | HAdV-C2 | 14-34/HAdV-C2 |
| FiCR | TCCAAGTTTAGTAATCAT | Fiber | HAdV-C2 | 1134-1117/HAdV-C2 |
| FiCL-2 | CCGCACCCACTATCTTCA | Fiber | HAdV-C1, HAdV-C5 | -29-12/HAdV-C1 |
| FiCR-2 | GTTCCAAGTTTGGGAATCAT | Fiber | HAdV-C1 | 1137-1117/HAdV-C1 |
| FiCR-3 | GTTCCTAGTTTAGGAACCAT | Fiber | HAdV-C5 | 1137-1117/HAdV-C5 |

^a^ Primer BL, BR, CDL, FiBL and FiBR were designed by Madich et al*.* (24).

^b^ Y refers to nucleobase cytosine or thymine.

^c^ R refers to nucleobase adenine or guanine.
