# Supplementary material for: High-Incidence of Human Adenoviral Co-Infections in Taiwan
Source: PLoS One. 2013 Sep 20;8(9):e75208. doi: 10.1371/journal.pone.0075208 (PMC3779158; doi:10.1371/journal.pone.0075208)
Supplement: Table S2 — Pairwise comparison of nucleotide and amino acid sequencesa. a About 1,500 nucleotides of hexon and 940 nucleotides of fiber genes from different types were analyzed. b NA, not available. (DOCX) [file pone.0075208.s003.docx]

Table S2. Pairwise comparison of nucleotide and amino acid sequences ^a^.

|  | Difference (%) | | | |
| --- | --- | --- | --- | --- |
|  | Hexon | | Fiber | |
| Types | Nucleotide | Amino acid | Nucleotide | Amino acid |
| HAdV-B3 | 0.0-0.2 | 0.0-0.7 | 0.0-0.1 | 0.0 |
| HAdV-B7 | 0.0-0.2 | 0.0-0.7 | 0.0-0.2 | 0.0-1.1 |
| HAdV-B11 | 0.0 | 0.0 | 0.0 | 0.0 |
| HAdV-C1 | 0.0-1.4 | 0.0-0.3 | 0.0-2.2 | 0.0-6.3 |
| HAdV-C2 | 0.0-0.9 | 0.0-0.3 | 0.0-1.3 | 0.0-2.1 |
| HAdV-C5 | 0.0-4.7 | 0.0-4.5 | 0.0-0.6 | 0.0-1.5 |
| HAdV-C6 | 0.0-0.2 | 0.0 | NA*^b^* | NA*^b^* |
| HAdV-E4 | 0.0 | 0.0 | 0.0-0.1 | 0.0-0.6 |

^a^ About 1,500 nucleotides of hexon and 940 nucleotides of fiber genes from different types were analyzed.

^b^ NA, not available.
